# Metabolic responses and benefits of glucagon‐like peptide‐1 (GLP‐1) receptor ligands

**DOI:** 10.1111/bph.15485

**Published:** 2021-05-10

**Authors:** Neil Tanday, Peter R. Flatt, Nigel Irwin

**Affiliations:** ^1^ Diabetes Research Group Ulster University Coleraine UK

**Keywords:** diabetes, GLP‐1, incretin, obesity

## Abstract

Glucagon‐like peptide‐1 (GLP‐1) is an incretin hormone that has undergone a revolutionary turnaround from discovery to clinically approved therapeutic. Rapid progress in drug design and formulation has led from initial development of short‐ and long‐acting drugs suitable for daily or weekly parenteral administration, respectively, through to the most recent approval of an orally active GLP‐1 agent. The current review outlines the biological action profile of GLP‐1 including the various beneficial metabolic responses in pancreatic and extra‐pancreatic tissues, including the gastrointestinal tract, liver, bone and kidney as well as the reproductive cardiovascular and CNS. We then briefly consider clinically approved GLP‐1 receptor ligands and recent advances in this field. Given the sustained evolution in the area of GLP‐1 drug development and excellent safety profile, as well as the plethora of metabolic benefits, clinical approval for use in diseases beyond diabetes and obesity is very much conceivable.

**LINKED ARTICLES:**

This article is part of a themed issue on GLP1 receptor ligands (BJP 75th Anniversary). To view the other articles in this section visit http://onlinelibrary.wiley.com/doi/10.1111/bph.v179.4/issuetoc

**Abbreviations**
ANP: atrial natriuretic hormone
ApoE: apolipoprotein E
CCK: cholecystokinin
FFA: free fatty acid
GIP: gastric inhibitory polypeptide
GLP‐1: glucagon‐like peptide‐1
TAP1/ABCD2: peptide transporter 1
SNAC: sodium *N*‐(8‐[2‐hydroybenzoyl] amino) caprylate

## 1 INTRODUCTION

**FIGURE 1:**
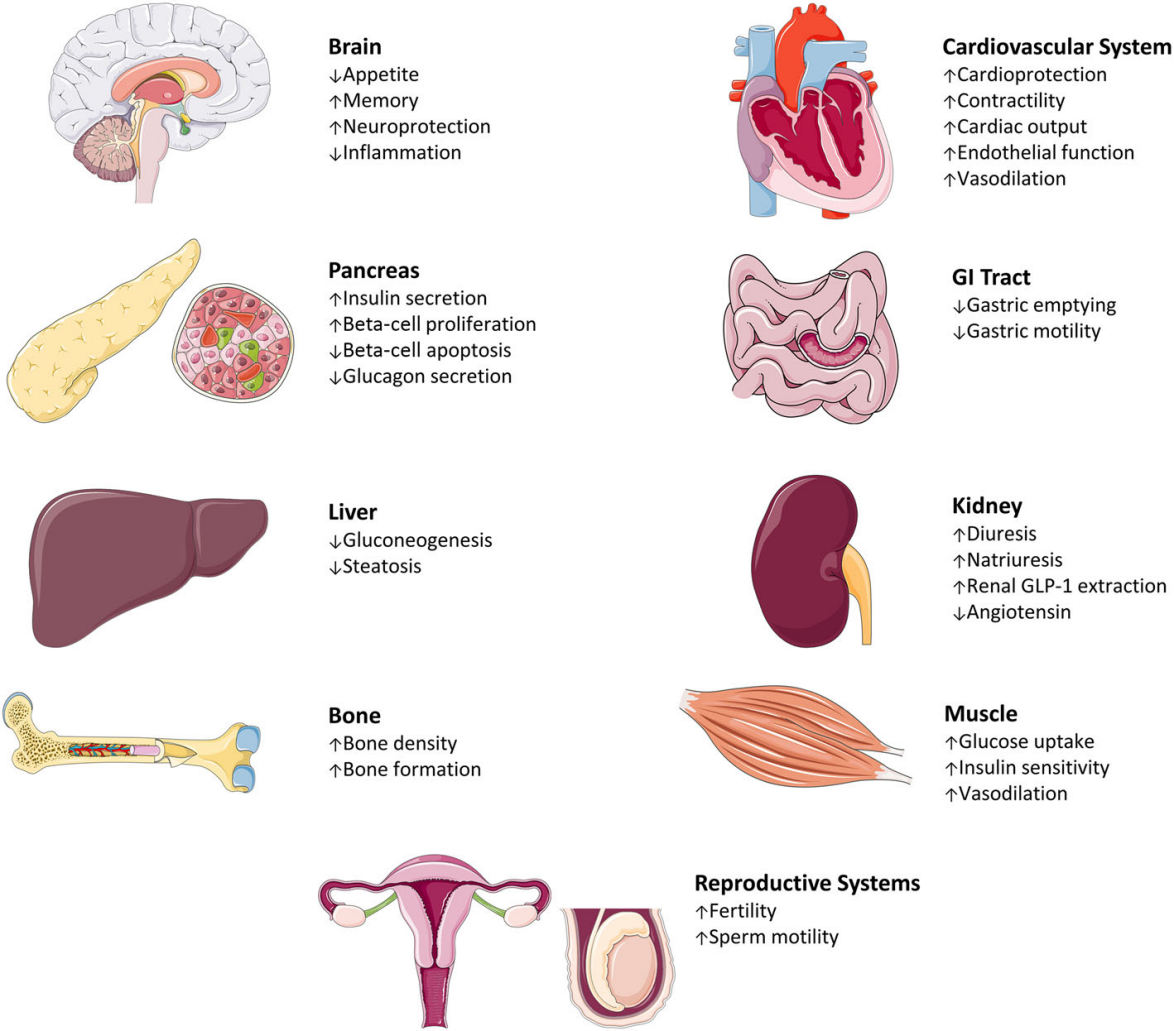
The metabolic actions of GLP‐1 across diverse organs including the pancreas, brain, gastrointestinal (GI) tract, liver, muscle, bone, kidney as well as the cardiovascular and reproductive systems

The physiological role of the gastrointestinal tract was traditionally thought to involve nutrient digestion and absorption, but it is now known to be the source of a plethora of peptide hormones involved in the regulation of metabolism and other body functions (Baggio & Drucker, 2007). Seminal work in the late 1960s led to the identification of peptide hormones with glucagon‐like immunoreactivity following gastrointestinal tract stimulation by glucose (Samols & Marks, 1967). Since then, two major gastrointestinal tract‐derived hormones involved in regulation of postprandial glucose have been identified, namely, glucagon‐like peptide‐1 (GLP‐1) and glucose‐dependent insulinotropic polypeptide (gastric inhibitory polypeptide; GIP), secreted from L‐cells and K‐cells of the gastrointestinal tract, respectively. Collectively, these two hormones account for 50%–70% of insulin secretion in response to a meal (Baggio & Drucker, 2007), with this action termed ‘the incretin effect’. Given the glucose‐dependent nature of GLP‐1 induced insulin secretion and retention of bioactivity in type 2 diabetes (Nauck, Kleine, et al., 1993), drugs based on the biological action of this hormone were rapidly translated to benefits in humans (Baggio & Drucker, 2007). Thus, the amino acid peptide sequence of GLP‐1 was first discovered by Habener and colleagues in the early 1980s through decoding of recombinant cDNA clones in anglerfish (Lund et al., 1982) and subsequently found to enhance insulin secretion in the perfused rat pancreas (Mojsov et al., 1987), with clinical approval of GLP‐1 mimetic for the treatment of type 2 diabetes mellitus (T2DM) following in 2005 (Kolterman et al., 2005). Although this original approval was largely based on the potent glucose‐dependent insulinotropic properties of GLP‐1 receptor (GLP‐1 receptor) activation on pancreatic beta‐cells, it is now clear that the GLP‐1 receptor is expressed on various other metabolically active tissues eliciting a range of biological effects across diverse organ systems (Figure 1).

## 2 GLP‐1 SECRETION

In terms of endogenous secretion, GLP‐1 producing L‐cells are predominantly located along the ileum and colon of the gastrointestinal tract (Eissele et al., 1992). With the apical surface of the L‐cell in contact with the gut lumen, GLP‐1 secretion is stimulated by the presence of intestinal nutrients (Eissele et al., 1992), albeit via distinct mechanisms. Thus, glucose absorption within the L‐cell leads to ATP production, subsequent closure of K_ATP_ channels and opening of voltage‐gated Ca^2+^ channels, a process known to be linked to sodium‐coupled glucose transporters (SGLT1) that sense ingested glucose (Parker et al., 2012). The resulting Ca^2+^ influx triggers exocytosis of GLP‐1 containing vesicles into the circulation. Alternatively, free fatty acids bind to and activate their respective free fatty acid L‐cell receptors, for example FFA1 (GPR40) and FFA4 (GPR120) to increase intracellular Ca^2+^ via Gq signalling pathways that stimulate PKC signalling leading to GLP‐1 secretion (Tolhurst et al., 2011). In particular, activation of the GPCR, bile acid (GPBA) receptor, increases L‐cell differentiation and elicits substantial GLP‐1 secretion (Lund et al., 2020).

In addition to this, proteins and amino acids are consistently shown to elicit L‐cell GLP‐1 secretion *in vitro* (Tolhurst et al., 2011), in rodents (Clemmensen et al., 2013) and in humans (Lejeune et al., 2006). This action is mediated by activation of Ca^2+^/calmodulin‐dependent kinase II (CaMKII), calcium‐sensing (CaS) receptor and peptide transporter 1 (TAP1 or ABCD2), leading to a rise in intracellular Ca^2+^ and subsequent GLP‐1 secretion (Diakogiannaki et al., 2013). Furthermore, intestinal L‐cells are situated in close proximity to enteric neurons and microvasculature, suggesting that GLP‐1 secretion is also influenced by neuronal and endocrine factors (Anini et al., 2002). As such, GIP and cholecystokinin (CCK‐8) are the two gut‐derived hormones implicated in GLP‐1 secretion. In rodents, intravenous treatment with GIP is associated with an increase in glucagon‐like immunoreactivity via a GIP‐GLP‐1 vagal axis Rocca and Brubaker (1999). Whether such a GIP‐GLP‐1 axis operates in humans is questionable, given that pharmacological doses of GIP fail to elicit GLP‐1 secretion in man (Mentis et al., 2011). In addition to these pharmacological stimuli, murine L‐cells display a circadian pattern of GLP‐1 secretion that peaks prior to the onset of feeding periods (Biancolin et al., 2020).

Contrary to extensive and growing knowledge around the stimulation of GLP‐1 secretion from enteroendocrine L‐cells, much less is known about potential mediators that provide feedback inhibition to GLP‐1 secretion. In this regard, the neuropeptide galanin has been shown to inhibit GLP‐1 secretion via action of the G_i_‐linked GAL_1_ receptor expressed on L‐cells (Psichas et al., 2015). Likewise, somatostatin (SRIF‐28) has been shown to inhibit GLP‐1 secretion, likely via modulation of the somatostatin 5 (SST_5_) receptor (Chisholm & Greenberg, 2002). Exploiting this knowledge, selective SST_5_ antagonists have been shown to augment circulating GLP‐1 levels in mice (Farb et al., 2017). Indeed, more recent evidence reveals that selective stimulation of colonic L‐cells leads to significant improvements in metabolic control, with obvious possible therapeutic implications (Lewis et al., 2020).

Once secreted, GLP‐1 has a short duration of biological action due enzymatic degradation by the ubiquitous enzyme dipeptidyl peptidase‐4 (DPP‐4) and efficient renal clearance, resulting in an *in vivo* t_½_ of around 5–10 min (Deacon et al., 1996). It is suggested that up to 75% of secreted GLP‐1 is degraded within the gut, with an additional 50% then degraded in the liver, before even entering the general circulation (Deacon et al., 1996). Within the circulation, GLP‐1 binds and activates the GLP‐1R expressed on various sites throughout the body. The GLP‐1 receptor is a membrane bound GPCR, coupled to Gαs that activates AC to increase cAMP and triggers intracellular cascades leading to various responses within each cell type (Mayo et al., 2003). However, there has been some controversy around the specificity of commercially available antibodies directed against the GLP‐1 receptor (Pyke & Knudsen, 2013), generating debate on the exact location of GLP‐1 receptor expression in the body (Pyke & Knudsen, 2013). Fortunately, the use of molecular biology techniques, alongside recent advances in monoclonal antibody development, has allowed for clearer identification of the GLP‐1 receptor, distinct from that of GLP‐2, GIP and glucagon receptors (Biggs et al., 2018; Pyke et al., 2014;). Further to this, the development of fluorescent probes, such as LUXendin645, allows for super‐resolution microscopic detection of the GLP‐1 receptor both *in vitro* and *in vivo* (Ast et al., 2020). As such, monoclonal antibodies with improved selectivity for GLP‐1 receptor have now been developed confirming true GLP‐1 receptor expression in the pancreas, brain, kidney, lung, heart and stomach (Pyke et al., 2014). Furthermore, transgenic mice expressing fluorescent markers in tissues that express the GLP‐1 receptor largely confirm these findings (Richards et al., 2014). In addition, mRNA expression of the GLP‐1 receptor has been observed in osteoblastic cell lines (Pacheco‐Pantoja et al., 2011), but there is limited evidence for presence of GLP‐1 receptor on human bone. Centrally, the GLP‐1 receptor is expressed within the following brain regions: cerebral cortex, +hypothalamus, hippocampus, thalamus, caudate‐putamen and globus pallidum (Alvarez et al., 2005). Finally, GLP‐1 receptor mimetic therapy has consistently shown to improve liver disease, possibly indirectly via anti‐inflammatory and weight‐reducing actions.

## 3 GLP‐1 AND THE ENDOCRINE PANCREAS

**FIGURE 2:**
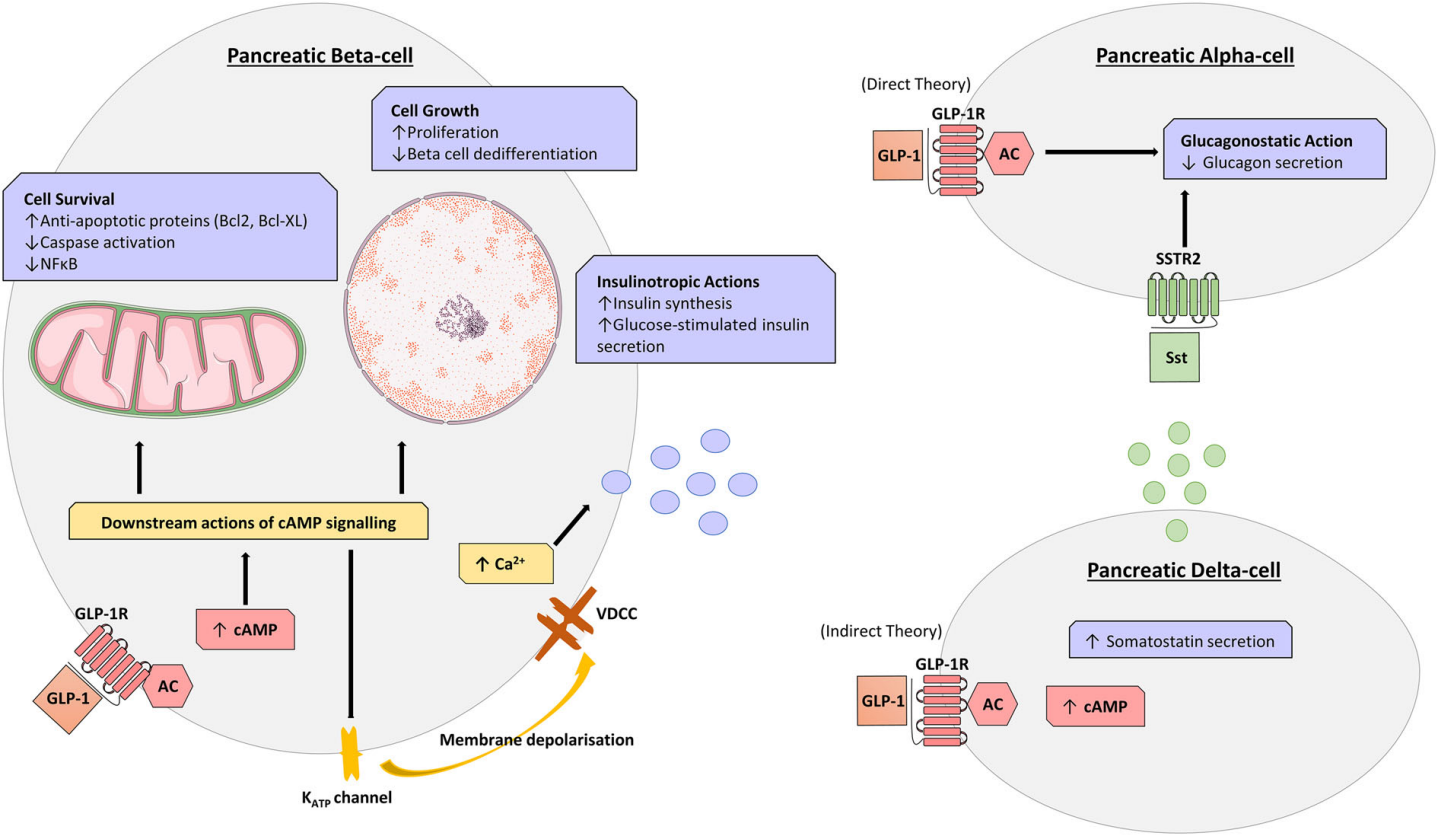
Actions of GLP‐1 within pancreatic alpha‐, beta‐ and delta‐cells. In beta‐cells, GLP‐1 binding to its receptor (GLP‐1R) triggers intracellular signalling cascades that positively influence insulin synthesis and secretion as well as beta‐cell proliferation and survival. The direct and indirect effects, mediated through the delta‐cell, of GLP‐1 receptor (GLP‐1R) activation on inhibition of alpha‐cell derived glucagon is also considered

Glucose‐stimulated insulin release from pancreatic beta‐cells is a tightly regulated process, that involves many complementary pathways. +In the case of GLP‐1, activation of the GLP‐1 receptor on beta‐cells triggers an intracellular signalling cascade that potentiates glucose‐stimulated insulin secretion, whilst also exerting more longer‐term benefits on beta‐cell growth and survival, ultimately leading to improvements in overall beta‐cell sensitivity and insulin production (Figure 2; Campbell & Drucker, 2013). Advances in islet cell lineage tracing technologies have also highlighted the importance of GLP‐1 receptor in maintaining beta‐cell identity and preventing beta‐cell de‐differentiation under situations of pancreatic islet stress (Tanday et al., 2020). In terms of the pancreatic alpha‐cell, GLP‐1 consistently suppresses glucagon secretion (Hare et al., 2009). Indeed, this glucagonostatic action is suggested to account for 50% of the blood glucose lowering ability of GLP‐1 (Hare, 2010). The exact mechanisms underlying this action are uncertain; however, there are two major theories (Figure 2). The ‘direct’ theory relies on alpha‐cells expressing the GLP‐1 receptor with GLP‐1 exerting a direct inhibitory action on alpha‐cells (De Marinis et al., 2010). However, even with the use of more specific antibodies and probes to accurately detect the GLP‐1 receptor, there are still conflicting reports on whether alpha‐cells express GLP‐1 receptor. Studies have demonstrated that only a small proportion, at most approximately 10%–12%, of mouse alpha‐cells express the GLP‐1 receptor (Ast et al., 2020), whereas others have failed to detect the GLP‐1 receptor on human alpha‐cells (Waser et al., 2015). Whether GLP‐1 receptor present on alpha‐cells make any meaningful contribution to the glucagonostatic effects of GLP‐1 receptor mimetics is questionable. Due to the lack of clear evidence regarding alpha‐cell GLP‐1 receptor expression, a second ‘indirect’ theory has emerged (Figure 2). This indirect theory ascribes to the idea that GLP‐1 mediates its glucagonostatic effect indirectly through stimulation of somatostatin secretion from pancreatic delta‐cells, that express functional GLP‐1 receptor (Ørgaard & Holst, 2017). In this regard, somatostatin consistently inhibits glucagon, insulin and GLP‐1 across all species. Given that GLP‐1 is known to stimulate delta‐cell secretions (Ørgaard & Holst, 2017), it is feasible that somatostatin exerts a paracrine inhibitory effect on neighbouring alpha‐cells. Indeed, in a perfused mouse pancreas model, administration of GLP‐1 suppressed glucagon secretion, with this inhibitory effect annulled in the presence of a specific somatostatin (SST_2_) receptor antagonist (Ørgaard & Holst, 2017). In similar fashion, the ability of liraglutide to reduce dapagliflozin‐induced hyperglucagonaemia is abolished in somatostatin receptor knockout mice (Saponaro et al., 2019). Collectively, these findings present strong evidence for the indirect theory of GLP‐1 mediated glucagon inhibition (Figure 2). In reality, GLP‐1 induced inhibition of glucagon secretion is a complicated process that requires further investigation, especially because GLP‐1 is known to stimulate release of amylin (Gedulin et al., 1997), GABA (Wendt et al., 2004) and zinc (Zhou et al., 2007), which can all independently modulate glucagon secretion. Nonetheless, the promotion of glucose‐dependent insulin secretion, coupled with reduced glucagon release, represents an ideal paradigm for diabetes therapy.

In contrast to GLP‐1 actions on alpha‐cells, the molecular actions underpinning potentiation of beta‐cell insulin secretion have been explored in depth (Figure 2). Upon GLP‐1 receptor binding and activation, the enzyme AC increases cAMP levels, which in turn stimulates PKA and Epac activity (Holz, 2004). PKA closes the beta‐cell ATP‐sensitive K^+^ (K_ATP_) channel to depolarise the cell membrane (Light et al., 2002). This depolarisation opens voltage‐dependant Ca^2+^ channels leading to increased Ca^2+^ influx, essential for exocytosis of insulin granules (MacDonald & Wheeler, 2003). In harmony with this, Epac proteins sensitise the K^+^ channel, lowering its ATP threshold for activation and further act on the endoplasmic reticulum to release Ca^2+^ cellular stores (Doyle & Egan, 2007). These pathways are critical for GLP‐1 mediated insulin secretory activity, as blocking cAMP accumulation (Härndahl et al., 2002) or PKA activity (Wang et al., 2001) eliminates GLP‐1 induced insulin secretion.

As well as stimulating glucose‐dependent insulin secretion, GLP‐1 exerts additional effects of pancreatic beta‐cells with obvious therapeutic benefits in diabetes. As such, GLP‐1 is able to slow the loss of beta‐cell mass in diabetes through its ability to increase proliferation (Arakawa et al., 2009) and protect against apoptosis (Li et al., 2003). Specifically, activation of PKA by GLP‐1 leads to an increase in pancreatic and duodenal homeobox 1, a transcription factor critical for maintenance of beta‐cell function and PKB (Akt) induced beta‐cell proliferation (Wang et al., 2001). However, it should be noted that adult human beta‐cells appear to have somewhat limited proliferative capacity, when compared with juvenile mouse or human cells (Dai et al., 2017), with important therapeutic implications. Interestingly, up‐regulation of beta‐cell pancreatic and duodenal homeobox 1 expression is also attributed to GLP‐1 mediated benefits on the maintenance of beta‐cell identity and prevention of beta‐cell de‐differentiation in situations of islet stress (Tanday et al., 2020). In addition to this, GLP‐1 receptor‐mediated intracellular beta‐cell signalling also leads to up‐regulation of anti‐apoptotic proteins, such as B‐cell lymphoma 2 (Bcl‐2) and B‐cell lymphoma‐extra large (BcL‐xL), as well as inhibition of caspase activation and NF‐κB to ultimately encourage beta‐cell survival and resistance to endoplasmic reticulum stress (Tsunekawa et al., 2007). Taken together, the compilation of these GLP‐1 induced benefits on pancreatic islet cells highlights the clinical benefits of GLP‐1 mimetics in diabetes.

### 3.1 GLP‐1 and the gastrointestinal tract

Activation of the GLP‐1 receptor within the CNS reduces gut contractility, slowing gastric motility and emptying (Goyal et al., 2019). By reducing gastric motility, nutrients are absorbed into the circulation at a slower rate, decreasing the postprandial spike in blood glucose (Smits et al., 2016), with obvious benefit in diabetes. The mechanism behind this action is multifaceted and involves vagal (parasympathetic) innervation, noradrenergic (sympathetic) innervation and NO signalling (Tolessa et al., 1998). This gastrointestinal tract effect is consistent across species, is observed in healthy and diabetic (Meier et al., 2003) humans and amplifies the antidiabetic actions of GLP‐1.

### 3.2 GLP‐1 and the cardiovascular system

Type 2 diabetes is strongly associated with increased cardiovascular disease risk (Marso, Bain, et al., 2016; Marso, Daniels, et al., 2016), and there has been recent strong emphasis on the ability current and future antidiabetic drugs to reduce cardiovascular disease mortality in diabetes (Marso, Bain, et al., 2016; Marso, Daniels, et al., 2016). Notably, the expression of GLP‐1 receptor has been detected within all four chambers of the heart, sinoatrial node and arteriole smooth muscle cells (Baggio et al., 2018; Pyke et al., 2014). In this regard, GLP‐1 infusion has been shown to improve endothelial function (Nystrom et al., 2004), with GLP‐1 mimetic therapy known to reduce arertial blood pressure (Sun et al., 2015) and offer overall cardiomyocyte protection (Asmar et al., 2017; Sjøberg et al., 2014; Wang et al., 2013). Collectively, these actions benefit cardiovascular health, as has been demonstrated in recent cardiovascular outcome trials using various GLP‐1 mimetics (Marso, Bain, et al., 2016; Marso, Daniels, et al., 2016). Specifically, in the SUSTAIN*‐*6 trial, patients with type 2 diabetes and cardiovascular disease risk had a reduced rate of cardiovascular death, non‐fatal myocardial infarction and non‐fatal stroke when treated with the recently approved GLP‐1 mimetic semaglutide (Marso, Bain, et al., 2016). Likewise, the LEADER trial concluded that a related GLP‐1 mimetic, namely, liraglutide, was beneficial in reducing the rate of non‐fatal myocardial infarction, stroke and first occurrence of death from cardiovascular causes (Marso, Daniels, et al., 2016). However, this cardiovascular disease benefit may be GLP‐1 mimetic specific, given that other trials using GLP‐1 mimetics with shorter half‐lives and lower homology to native GLP‐1, such as lixisenatide (ELIXSA) or exenatide (EXSCEL), failed to show a significant cardiovascular disease benefit (Holman et al., 2017). Despite this variation, a more recent meta‐analysis of all these trials confirmed the beneficial actions of GLP‐1 mimetics through a 13% relative risk reduction in cardiovascular disease mortality, 12% risk reduction in all‐cause mortality and 10% relative risk reduction in cardiovascular death, non‐fatal myocardial infarction and non‐fatal stroke (Bethel et al., 2018). Ultimately, clinical trials such as these have confirmed that, similar to sodium/glucose cotransporters 2 (SGLT2) inhibitors, GLP‐1 mimetics have established cardiovascular disease benefits in diabetes.

The mechanisms underpinning the cardiovascular benefit of GLP‐1 mimetics are multifaceted. GLP‐1 mimetics can reduce traditional cardiovascular disease risk factors such as obesity, whilst also exerting anti‐inflammatory and anti‐atherosclerotic effects as well as having positive direct modulatory effects on endothelial, cardiac and renal function (Garg et al., 2019). In type 2 diabetes mellitus, GLP‐1 receptor activation has been shown to reduce arterial blood pressure, due to a direct vasodilatory action combined with indirect actions on lowering body weight and inducing kidney natriuresis (Asmar et al., 2019). In all four major cardiovascular outcome trials, namely SUSTAIN‐6, LEADER, ELIXA, EXCSEL, systolic BP was significantly reduced with GLP‐1 mimetic therapy, with the greatest mean reduction (5.4 mmHg) associated with 1 mg once‐weekly semaglutide treatment (Marso, Bain, et al., 2016). Similarly, all four trials highlighted GLP‐1 mimetic induced weight loss, again with the greatest effect (4.3 kg weight loss) observed in the semaglutide treated group (Marso, Bain, et al., 2016). GLP‐1 mimetics, such as liraglutide and exenatide, have also been shown to reduce LDL cholesterol, total cholesterol and triglyceride levels (Sun et al., 2015). Collectively, these actions account for at least some of the cardiovascular disease benefits observed with GLP‐1 mimetic therapy.

Given that the GLP‐1 receptor is expressed on all four chambers of the heart as well as the sinoatrial node, it is likely that GLP‐1 and its mimetics exert a direct action on cardiac cells (Baggio et al., 2018). In agreement with this, GLP‐1 therapy has a protective effect on cardiomyocytes during myocardial infarction in mice (Nikolaidis et al., 2004). Interestingly, this beneficial action is still present in cardiac‐specific GLP‐1 receptor knockout mice, implying possible important indirect effects (Ussher et al., 2014). GLP‐1 and associated mimetics have also been shown to increase heart rate (Robinson et al., 2013) via direct receptor mediated actions (Baggio et al., 2017), but this effect was variable between mimetics. As such, in a head‐to‐head study with lixisenatide and liraglutide, the shorter‐acting agent lixisenatide produced a modest, transient 1–3 beat per minute increase in HR, whilst the longer acting GLP‐1 mimetic liraglutide was associated with a more distinct and sustained 6–10 beats per minute elevation (Meier et al., 2015). The potential impact of elevated heart rate in patients with heart failure does need to be carefully considered (Marso, Bain, et al., 2016). Furthermore, heart rate is well known to increase postprandially, and GLP‐1 mimetic mediated elevations of heart rate could also be a compensatory consequence of GLP‐1‐induced vasodilation in specific tissues (Asmar et al., 2017), but such a GLP‐1 mimetic effect still needs to be confirmed.

### 3.3 GLP‐1 and inflammation

**FIGURE 3:**
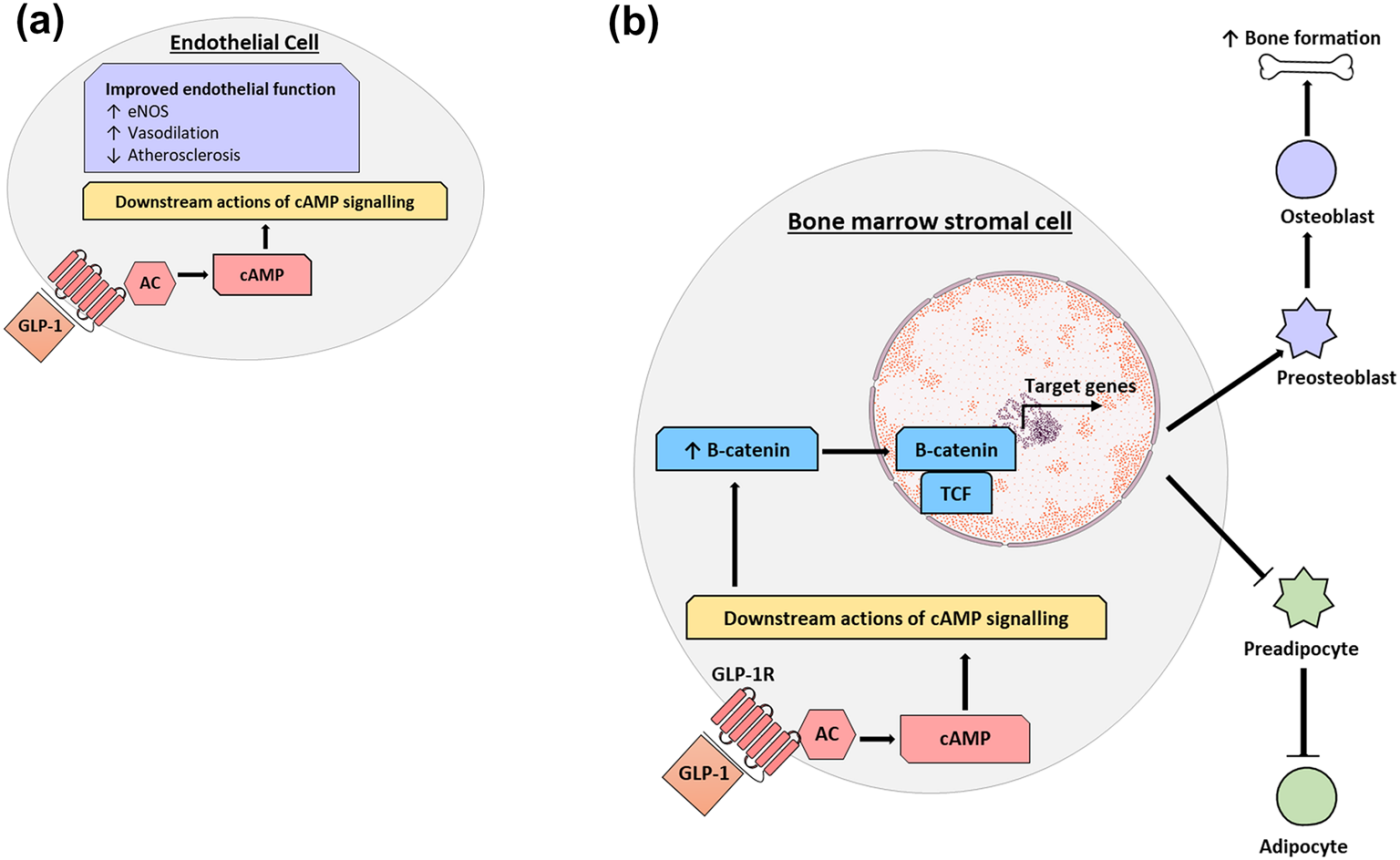
Actions of GLP‐1 on (a) endothelial cells and (b) bone. (a) GLP‐1 triggers a signalling cascade inside endothelial cells to mediate vasodilation and reduce atherosclerosis, to collectively improve cardiovascular health. (b) GLP‐1 binds to its receptor (GLP‐1R) on bone marrow stromal cells to activate intracellular signalling cascades that prevent the breakdown of B‐catenin. This augments gene expression to favour differentiation of the bone marrow stromal cell into pre‐osteoblasts, rather than pre‐adipocytes, and increase bone formation

Further to this, GLP‐1 therapy has also been shown to augment anti‐inflammatory and anti‐atherosclerotic processes, demonstrated by their ability to reduce occurrence of myocardial infarctions and strokes (Tanaka & Node, 2018). In this regard, GLP‐1 mimetics have been shown to impede inflammatory responses and reduce atherosclerosis development (Figure 3a). Specifically, in animal models of atherosclerosis, namely, ApoE^−/−^ and LDL receptor^−/−^ knockout mice, GLP‐1 treatment reduced plaque size (Bjørnholm et al., 2020). The GLP‐1 mimetics exendin‐4 and semaglutide have also been shown to reduce cerebrovascular infarct size in rodent models of cerebral ischaemia (Basalay et al., 2019). In patients with acute myocardial infarction, administration of GLP‐1 or its mimetics improved ventricular function and reduced reperfusion injury (Nikolaidis et al., 2004). Improvement of endothelial dysfunction is another mechanism through which GLP‐1 mimetics exert their cardiovascular disease benefit (Nystrom et al., 2004). As such, liraglutide is known to ameliorate vascular endothelial dysfunction via suppression of oxidative stress and direct promotion of endothelial‐derived NOS (eNOS) mediated NO production and vasodilation (Figure 3a; Li et al., 2020). However, it should be noted that others have failed to observe clear beneficial effects of GLP‐1 mimetics on endothelial function (Faber et al., 2015).

GLP‐1 and associated mimetics also have established anti‐inflammatory benefits beyond cardiovascular disease (Lee & Jun, 2016). As such, intraepithelial lymphocytes (IELs) of the gastrointestinal tract express GLP‐1 receptor, with activation resulting in reduced cytokine production to positively control innate immunity and gut barrier function (Yusta et al., 2015). In this regard, liraglutide improves aspects of inflammatory skin diseases, such as psoriasis, through beneficial anti‐inflammatory actions on immune cells (Hogan et al., 2011). Likewise, liraglutide has been shown to reduce lung fibrosis in a bleomycin‐induced rodent model of lung disease, by directly decreasing the expression of pro‐fibrotic cytokines markers (Fandiño et al., 2020). Collectively, these studies highlight the potential of GLP‐1 mimetics for the treatment of diseases and disorders driven by chronic inflammation.

### 3.4 GLP‐1 and the kidney

GLP‐1 and associated mimetics elicit actions on the renal system, but effects may be dependent on species studied, renal health status and concentration of GLP‐1 mimetic employed (Hviid & Sørensen, 2020). In rodents, GLP‐1 acts on kidney GLP‐1 receptors located on renal vascular smooth muscle (Pyke et al., 2014), to increase renal plasma flow and glomerular filtration rate as well as diuresis and natriuresis whilst reducing renal inflammation, fibrosis and oxidative stress, via cAMP and PKA signalling pathways (Lee & Jun, 2016). The presence of SGLT1 on L‐cells of the gastrointestinal tract (Parker et al., 2012) may also represent sodium sensing capability within the gut and suggest important gut‐kidney crosstalk in relation to regulation of ingested sodium (Asmar et al., 2020). Tensive status also seems to be an important factor in determining the renal actions of GLP‐1. As such, when compared with normotensive rats, hypertensive rats exhibit reduced renal GLP‐1 receptor expression and related effects (Ronn et al., 2017). In addition, renal output is closely associated with cardiac function, with GLP‐1 receptor activation at both sites determining overall responses. In humans, GLP‐1 infusion has recently been shown to exert a natriuretic effect as well as suppress angiotensin II release independent of renal plasma flow and glomerular filtration rate (Asmar et al., 2021), but the specific site of GLP‐1 receptor expression required for these effects still to be determined. Alternatively, in rodents, a GLP‐1/atrial natriuretic peptide (ANP) axis exists, whereby activation of the GLP‐1 receptor in cardiac tissue stimulates ANP secretion to evoke renal natriuresis and reduced BP (Kim et al., 2013). GLP‐1 has also been shown to exert positive actions on the renal system in the treatment of both diabetic and non‐diabetic kidney disease (Roscioni et al., 2014). In addition to cytoprotective and anti‐inflammatory actions, GLP‐1 also acts to increase renal plasma flow, glomerular filtration rate, renal interstitial fluids and urinary flow rate, whilst reducing tubular necrosis (Skov et al., 2013). The importance of GLP‐1 receptor action within the kidney is exemplified in GLP‐1 receptor knockout mice that display increased renal oxidative stress (Fujita et al., 2014). Together, these actions highlight the potential of GLP‐1 receptor mimetics for treating kidney disease and improving kidney function in diabetes.

### 3.5 GLP‐1 and bone

Type 2 diabetes mellitus is associated with increased bone fracture risk, but the mechanisms behind this effect are still to be fully elucidated (Mabilleau, Pereira, & Chenu, 2018). Diabetic animal models present with a loss of bone mineral density, which can be restored through administration of GLP‐1 receptor mimetics (Mansur et al., 2015, 2019a). In addition, exenatide has been shown to stimulate osteoblast activation and restore bone formation in an ovariectomy‐induced model of bone loss (Mabilleau et al., 2013). Whether these beneficial effects are linked to direct activation of GLP‐1 receptor on bone remains to be determined, given conflicting reports GLP‐1 receptor expression on bone (Jeon et al., 2014). One theory suggests that GLP‐1 acts on bone marrow stromal cells, with transcription of genes to promote osteoblast differentiation and inhibit adipocyte differentiation to ultimately favour bone formation, although this concept and presence of GLP‐1 receptor on marrow stromal cells still requires further clarification (Figure 3b). GLP‐1 receptor mediated improvements in bone strength and quality have also been demonstrated in various distinct forms of diabetes including insulin‐deficient type 1 diabetic mice (Mansur et al., 2015), insulin‐resistant high fat fed diabetic mice (Mansur et al., 2019a) as well as genetically induced type 2 diabetic animal models (Sun, Lu, et al., 2016). The observed positive actions of sitagliptin on bone are likely due to the elevations of both GLP‐1 and GIP levels (Mansur et al., 2019b), given that GIP has well documented benefits on bone in animals and humans (Gobron et al., 2020; Mabilleau et al., 2016; Mabilleau, Gobron, et al., 2018; Stensen et al., 2020; Vyavahare et al., 2020). In the clinic, recent reports show exenatide to have no impact on bone fractures, whereas lixisenatide and liraglutide reduce fracture occurrence (Cheng et al., 2019).

### 3.6 GLP‐1 and liver

Despite conflicting reports on whether hepatocytes express the GLP‐1 receptor (Gupta et al., 2010; Pyke et al., 2014), GLP‐1 has been shown to positively impact hepatic gluconeogenesis, glycogen synthesis and glycolysis (Gupta et al., 2010). In this regard, the impact of GLP‐1 mimetics on liver function is likely to be linked to activation of GLP‐1 receptor on immune macrophages to attenuate T‐cell mediated inflammation (Nagashima et al., 2011). In disease states, GLP‐1 receptor mimetics reduce hyperlipidaemia, liver fibrosis and inflammation in non‐alcoholic fatty liver disease (NAFLD) (Armstrong et al., 2016; Newsome et al., 2020) as well as liver fat content in type 2 diabetes (Petit et al., 2017). Similarly, in animal models, GLP‐1 also imparts beneficial effects on the liver, with exendin‐4 reducing oxidative stress and improving hepatic steatosis and inflammation in diabetic and atherosclerotic animal models, respectively (Sharma et al., 2011). In animal models of both acute and chronic liver injury, liraglutide protected against hepatotoxicity, associated with a reduction in oxidative stress, improved liver mitochondrial function and insulin resistance (Guo et al., 2018; Wang et al., 2017). Further research is required to demonstrate whether these hepatic benefits are mediated through direct GLP‐1 receptor action on hepatocytes or indirectly though GLP‐1 receptor induced weight loss, reduction in HbA1c and augmented lipid metabolism and insulin sensitivity.

### 3.7 GLP‐1 and fertility

Gut hormones, including GLP‐1, have been shown to impact the reproductive system and effect fertility (Moffett & Naughton, 2020). Thus, GLP‐1 receptor signalling increases menstrual frequency and chance of pregnancy in women with polycystic ovary syndrome (PCOS) (Liu et al., 2017). Additional actions have been identified in animal studies showing that GLP‐1 mimetics can restore ovarian morphology (Sun, Ji, et al., 2016) and improve development of ovarian follicles (Yang & Wang, 2016). Moreover, GLP‐1 mimetics can reduce testicular inflammation, leading to improved sperm motility and activity in diet‐induced obese mice (Zhang et al., 2015). Further to this, and although not directly related to GLP‐1 receptor mediated effects on fertility, the expansion in beta‐cell mass that occurs during pregnancy is linked to pancreatic alpha‐cell production of GLP‐1, which exerts a positive paracrine effect on neighbouring beta‐cells to encourage growth and proliferation (Moffett et al., 2014). In harmony with these findings, GLP‐1 receptor knockout mice exhibit delayed puberty, irregular oestrus cycles, impaired fertility and reduced litter sizes (MacLusky et al., 2000). The actions of GLP‐1 on fertility have yet to be fully exploited in the clinic, and further research is required to develop a suitable treatment options in respect to polycystic ovary syndrome and infertility.

### 3.8 GLP‐1 and the brain

The GLP‐1 receptor is expressed throughout many regions of the brain including the brainstem, cerebellum, cerebral cortex, hippocampus, hypothalamus, substantia nigra and thalamus (Cork et al., 2015). As a result, GLP‐1 receptors have important and potential pharmacologically exploitable effects within the CNS. The discussion of GLP‐1 receptor mediated CNS actions, including aspects of neuroprotection, hypothalamic regulation of food intake, stress response as well as locally produced GLP‐1, is largely outside the scope of our current review and is covered in detail within other reviews in this themed issue.

## 4 CLINICALLY APPROVED GLP‐1 RECEPTOR LIGANDS

The extensive biological action profile of GLP‐1 detailed above, with notable benefits in various disease states, promotes the wide therapeutic use of enzymatically stable, longer acting GLP‐1 analogues. However, to date, the use of GLP‐1 receptor‐based therapies has only been approved in the treatment of obesity and diabetes. In this regard, GLP‐1 receptor drugs can be subdivided pharmacologically by their duration of action into short‐acting and long‐acting classes (Aroda, 2018). Short‐acting GLP‐1 receptor ligands, namely, exenatide and lixisenatide, provide shorter elevations in circulating GLP‐1 levels (2–3 h) that act quickly to delay gastric emptying and reduce postprandial blood glucose levels (Nauck et al., 2011). Whereas long‐acting GLP‐1 receptor ligands, namely, liraglutide, albiglutide, dulaglutide and exenatide‐LAR, lead to more prolonged periods of GLP‐1 receptor activation (>24 h) to reduce fasting blood glucose (Buse et al., 2009). This more consistent elevation in plasma GLP‐1 levels, and therefore potential for an uninterrupted receptor activation profile, appears to result in greater improvements in HbA1c levels when compared with short‐acting GLP‐1 compounds (Buse et al., 2009). However, longer acting GLP‐1 receptor ligands have been shown to induce some tachyphylaxis and as such have a more limited impact on gastric motility and are therefore unable to reduce postprandial hyperglycaemia as effectively as their short‐acting counterparts (Nauck et al., 2011). Both short and long‐acting GLP‐1 receptor ligands induce weight loss, confirming that this action is not secondary to delaying gastric motility but rather due to direct actions within the CNS and hypothalamus. In addition, although highly likely, it is still unknown whether a more consistent GLP‐1 receptor activation profile is observed with longer acting GLP‐1 receptor mimetics.

### 4.1 Early progress with clinically approved GLP‐1 receptor mimetics

**FIGURE 4:**
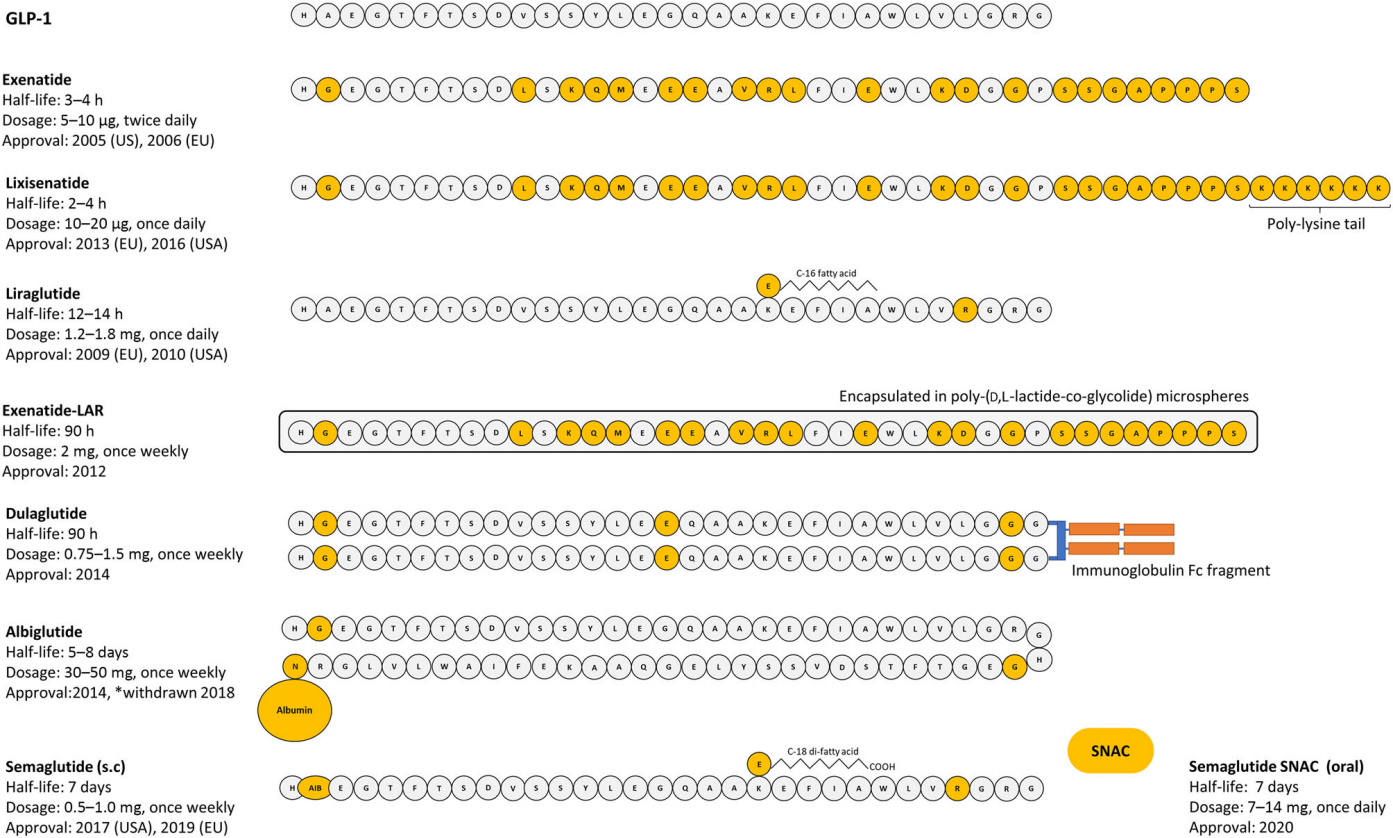
Clinically approved GLP‐1 mimetics prescribed for the treatment of diabetes. Structural modifications of GLP‐1 mimetics, compared with native GLP‐1, are highlighted in gold. Information on drug half‐lives, dosage and date of initial approval is also included. *Withdrawn from the market in 2018

The progress with developing new and enhanced clinically approved GLP‐1 receptor mimetics has been frequent over the years (Figure 4). As such, exenatide was the first GLP‐1 receptor ligand drug approved for type 2 diabetes in 2005 as a twice‐daily preparation (Nielsen et al., 2004) and was swiftly followed by approval of once‐daily liraglutide (Drucker et al., 2010). Exenatide extended‐release (exenatide‐LAR) was the first approved once‐weekly GLP‐1 receptor mimetic in 2012, followed by albiglutide, dulaglutide and semaglutide (Dhillon, 2018), with lixisenatide also gaining approval in 2016 as another once‐daily administered drug option (Heimbürger et al., 2019). It should however be noted that albiglutide was globally withdrawn from the market in July 2018 for economic reasons. Nonetheless, all these GLP‐1 have proven clinical effectiveness, but each requires parenteral administration due to peptidic nature of GLP‐1, with obvious patient compliance issues. To date, liraglutide is the only GLP‐1 mimetic approved for the treatment of obesity, albeit at a slightly increased dose than that used for diabetes therapy (Figure 4).

### 4.2 Recent advance with GLP‐1 receptor mimetics

Semaglutide represents the most recently approved once‐weekly formulation, first gaining clinical approval in 2017. Semaglutide is composed of human GLP‐1 molecule with C‐18 acylation at Lys^26^ and, amino acid substitution of Ala^2^ with Abu^2^ to impart full DPP‐4 resistance and an additional Lys^34^ for Arg^34^ amino acid replacement (Buckley et al., 2018). Collectively, these alterations result in a biological half‐life of approximately 7 days allowing for once‐weekly administration (Dhillon, 2018). More strikingly, recent advances in peptide formulation and delivery led to the generation of a semaglutide drug that that can be delivered orally. As such, oral semaglutide (Rybelsus) was FDA approved in 2019 (with EMA approval in early 2020) as the first non‐injectable GLP‐1 mimetic suitable for once‐daily oral administration in humans (Hedrington & Davis, 2019). Orally active semaglutide is formulated with an absorption enhancer, namely, sodium *N*‐(8‐[2‐hydroybenzoyl] amino) caprylate (SNAC), to encourage transcellular absorption of intact semaglutide through the gastric membrane by causing a localised increase in pH (Buckley et al., 2018). This represents a major milestone for GLP‐1 therapeutics and will likely herald further unprecedented progress in this field. As a monotherapy, oral semaglutide promotes dose‐dependent reductions in HbA1c and weight loss (Aroda et al., 2019). Compared with injectable liraglutide, oral semaglutide was equally effective at reducing HbA1c during a 26 week treatment regimen and actually exhibited superior efficacy over liraglutide following 52 weeks treatment (Pratley et al., 2019). Moreover, in a similar head‐to‐head comparison (SUSTAIN‐10), oral semaglutide was superior to liraglutide in reducing weight and HbA1c but was associated with gastrointestinal adverse events (Capehorn et al., 2020). In that regard, gastrointestinal tract‐related side effects associated with oral semaglutide resulted in 6%–7% of patients discontinuing during these trials (Aroda et al., 2019; Pratley et al., 2019), but this is similar to other previously approved GLP‐1 mimetics. Further to its clinical use in diabetes, once‐weekly injection of 2.4 mg semaglutide has shown significant promise as an anti‐obesity agent by reducing body weight by up to 15% in overweight adults (Wilding et al., 2021). Additional clinical trials, namely, PIONEER 11 and PIONEER 12, are currently ongoing to assess the safety and efficacy of oral semaglutide monotherapy or when combined with sitagliptin respectively, with data expected in 2021.

Other recent notable advances in the area of GLP‐1 therapy relate to simultaneously supplementing GLP‐1 receptor signalling with activation of receptors for related hormones, which exhibit complementary mechanisms of action (Irwin & Flatt, 2009a). The most obvious companion for GLP‐1 in this regard is its sister incretin hormone GIP (Stumvoll & Tschöp, 2018). Thus, like GLP‐1, GIP exhibits prominent glucose‐dependent insulinotropic actions in addition to numerous other beneficial extrapancreatic glucose‐lowering actions (Irwin & Flatt, 2015). Initially, the hypoglycaemic effectiveness of GIP was believed to be severely impaired in patients with type 2 diabetes mellitus (Nauck, Heimesaat, et al., 1993), with preclinical studies in animal models of diabetes revealing limited additive positive effects of combination therapy using long‐acting, enzymatically stable GIP and GLP‐1 compounds (Irwin, McClean, Cassidy, et al., 2007; Irwin, McClean, & Flatt, 2007). However, clinical studies clearly demonstrated that GIP insensitivity in type 2 diabetes mellitus is surmountable (Højberg et al., 2009), suggesting potential for additive antidiabetic benefits of GIP alongside GLP‐1 in humans. This area of research was ultimately brought to the fore by the generation of single peptide molecules capable of co‐activating GIP and GLP‐1 receptors, dubbed the dual‐acting ‘twincretin’ unimolecular drugs (Finan et al., 2013; Gault et al., 2013). One such dual‐acting drug developed by Lilly, namely, tirzepatide, with bias towards the GIP receptor (over GLP‐1 receptor (Coskun et al., 2018), is currently in Phase 3 clinical trials. In this regard, tirzepatide appears to exert remarkable positive effects on glycaemic control and body weight loss in type 2 diabetes mellitus, with benefits well beyond that observed in patients treated with GLP‐1 receptor mimetic therapy alone (Frías, 2020).

Interestingly, there is also a suggestion that inhibition of GIP receptor signalling can induce benefits in obesity and related diabetes (Irwin & Flatt, 2009b). As such, activation of GIP receptor leads to accumulation of lipids in peripheral tissues (Irwin et al., 2020). It follows that blockade of GIP receptor action could counter insulin resistance and improve metabolic status through prevention of fat deposition. Indeed, a recent observation reveals that sustained GIP receptor agonism actually leads to desensitisation of the GIP receptor to impart metabolic benefits (Killion et al., 2020). The therapeutic benefits of combined GLP‐1 receptor agonism and GIP receptor antagonism have also been investigated, with largely positive outcomes observed in preclinical studies (Irwin et al., 2009; Killion et al., 2018). Encouragingly, several other dual‐, or even triple‐acting, compounds with a GLP‐1 backbone have been produced, and many of these reveal clear metabolic benefits over GLP‐1 receptor agonism alone (Bhat et al., 2013; Hasib et al., 2018; Irwin et al., 2015; Jall et al., 2017; Khajavi et al., 2018; Pathak et al., 2018). In brief, it appears that combinatorial unimolecular therapies, which incorporate GLP‐1 receptor benefits together with the metabolic advantages of other related gastrointestinal tract‐derived hormones, have unmistakeable therapeutic potential for obesity, diabetes and beyond.

## 5 CLOSING REMARKS

It is somewhat hard to fathom that a single gut‐derived hormone like GLP‐1 can exert such significant beneficial actions across multiple organ systems, with clear therapeutic potential. Correctly utilising this hormone to take full advantage of all such biological actions has the potential to treat multiple pathologies and provide benefit to many patients. To date, approval for use of GLP‐1 mimetics has only be gained in diabetes and obesity. However, additional positive effects of GLP‐1 receptor activation in the gastrointestinal tract, liver, bone and kidney as well as the reproductive, cardiovascular and central nervous systems, whether direct or indirect, suggests further readily exploitable clinical potential. Finally, significant advancements in the pharmaceutical development of GLP‐1‐based drugs, leading from initial generation of injectable short‐ and long‐acting mimetics to now orally active GLP‐1 receptor ligands, opens up the therapeutic benefits of this class of drugs to a much wider cohort of patients. It is clear that GLP‐1 receptor mimetics have had a dramatic and positive impact on diabetes and obesity treatment regimens within a relatively short time period, and we await further progress on the therapeutic utility of GLP‐1‐based drugs with real optimism. This may ultimately involve exploitation with other metabolically active gut hormones in the form of unimolecular dual or triple acting receptor agonists.

### 5.1 Nomenclature of targets and ligands

Key protein targets and ligands in this article are hyperlinked to corresponding entries in the IUPHAR/BPS Guide to PHARMACOLOGY http://www.guidetopharmacology.org and are permanently archived in the Concise Guide to PHARMACOLOGY 2019/20 (Alexander et al., 2019).

## AUTHOR CONTRIBUTIONS

All authors contributed equally to conception and design, analysis and interpretation of data. N.T. drafted the manuscript, with P.R.F. and N.I. revising it critically for important intellectual content. All authors approved the final version of the manuscript.

## CONFLICT OF INTEREST

N.I. and P.R.F. hold patents for exploitation of gut peptide based therapeutics. P.R.F. also serves on scientific advisory boards and has received speaker's honoraria and research support from several companies with interests in glucose‐lowering drugs and incretin‐based therapies. N.T. declares no conflict of interest.

## Data Availability

Data sharing is not applicable to this article because no new data were created or analysed in this study.
